# Hummingbird ingestion of low-concentration ethanol within artificial nectar

**DOI:** 10.1098/rsos.230306

**Published:** 2023-06-21

**Authors:** Julia Choi, Lilianne Lee, Aleksey Maro, Ammon Corl, Jimmy A. McGuire, Rauri C. K. Bowie, Robert Dudley

**Affiliations:** ^1^ Department of Integrative Biology, University of California, Berkeley, CA 94720, USA; ^2^ Smithsonian Tropical Research Institute, Balboa, Republic of Panama

**Keywords:** bird, ethanol, flower, mutualism, nectar, pollination

## Abstract

Both frugivores and nectarivores are potentially exposed to dietary ethanol produced by fermentative yeasts which metabolize sugars. Some nectarivorous mammals exhibit a preference for low-concentration ethanol solutions compared to controls of comparable caloric content, but behavioural responses to ethanol by nectar-feeding birds are unknown. We investigated dietary preference by Anna's Hummingbirds (*Calypte anna*) for ethanol-enhanced sucrose solutions. Via repeated binary-choice experiments, three adult male hummingbirds were exposed to sucrose solutions containing 0%, 1% or 2% ethanol; rates of volitional nectar consumption were measured over a 3 h interval. Hummingbirds did not discriminate between 0% and 1% ethanol solutions, but exhibited significantly reduced rates of consumption of a 2% ethanol solution. Opportunistic measurements of ethanol concentrations within hummingbird feeders registered values peaking at about 0.05%. Ethanol at low concentrations (i.e. up to 1%) is not aversive to Anna's Hummingbirds and may be characteristic of both natural and anthropogenic nectars upon which they feed. Given high daily amounts of nectar consumption by hummingbirds, chronic physiological exposure to ethanol can thus be substantial, although naturally occurring concentrations within floral nectar are unknown.

## 1. Background

Nutritional choices during foraging can be influenced by many factors, including elemental composition of food relative to stoichiometric balance, macro- versus micronutrient representation and the presence of toxins (see [1–3]). Many fruits and floral nectars are attractive to animals because of a high sugar content and thus caloric benefit. However, their consumption may also lead to the ingestion of ethyl alcohol (henceforth, ethanol), which is a potential toxin at high dosages but can potentially be beneficial at low levels (i.e. via hormesis; see [4]). Carbohydrates within fruit and nectar can naturally ferment as a consequence of the common presence of fermentative yeasts, which anaerobically metabolize simple sugars to yield ethanol. Ethanol concentrations within nectar of bird-visited flowers are however unstudied in this regard, in spite of the major global radiations of avian nectar-feeders (e.g. the species-rich families Meliphagidae, Nectariniidae and Trochilidae). By contrast, ethanol content for a mammal-consumed nectar from an Indo-Malayan palm species can be as high as 3.8% [5], although broad surveys of ethanol within flowers have never been carried out.

Ethanol moreover may have multiple roles in nectarivore sensory and feeding biology. For example, associated vapour plumes could serve as an olfactory signal of caloric availability, and the gustatory sensation of ethanol may stimulate feeding and enhance net caloric gain (see [4]). Gochman *et al*. [6] showed that aye-ayes (*Daubentonia madagascariensis*) and slow lorises (*Nycticebus coucang*) prefer to consume solutions of higher over lower concentrations of ethanol (at levels up to 5% ethanol for the aye-aye, and 4% for the slow loris). In addition, Geoffroy's spider monkeys (*Ateles geoffroyi*) presented with a binary choice between a sucrose solution with no ethanol and a solution with concentrations of 0.1%, 0.5% or 3% ethanol preferred the higher concentration solution [7]. These and related studies (e.g. [8,9]) falsify the hypothesis of Janzen [10] that naturally occurring dietary ethanol is necessarily aversive to vertebrates. However, most results to date pertain to mammals and are of limited taxonomic representation; further studies across a wider range of species are required to assess whether low-level ethanol within food either attracts or deters animal consumers.

Nectar-feeding is widespread among birds [11,12], and the microbial flora within nectar often includes fermentative yeast [13–16]. Sugar solutions within artificial hummingbird feeders may also ferment, given microbial inoculation from birds and other animal vectors [17]. The possibility for chronic exposure to ethanol is thus substantial for nectar-feeding birds, and particularly for obligate nectarivores, given their high daily rates of fluid consumption. Even very low concentrations of ingested ethanol could, as accumulated through a day's feeding, result in a substantial dosage. Here, we describe the results of binary-choice experiments evaluating behavioural preference of Anna's Hummingbirds (*Calypte anna*) for sucrose solutions with varying ethanol content and also their choices between ethanol-free and ethanol-containing solutions. We hypothesize that, given the likely natural occurrence of nectar fermentation, low-concentration ethanol would be tolerated by hummingbirds. We also measured ethanol concentrations within hummingbird feeders to assess potential consumption associated with feeding from such devices.

## 2. Methods

Three adult male Anna's Hummingbirds, with an average (s.d.) body mass of 4.48 (0.35) g, were captured from the wild and were housed individually for up to a month. Birds were provided a standard nectar-feeding diet (Nektar Plus, Nekton GmbH, Pforzheim, Germany), made daily at a concentration of approximately 20% v/v in deionized water. This synthetic nectar solution was provided ad libitum via 10 ml syringes with plastic flowers attached to the 2 mm opening of the syringe. Birds were habituated to this diet for a minimum of 2 days before experiments.

Aqueous solutions of 1% and 2% ethanol in 20% sucrose solution (v/v) were made using reagent-grade 200 proof anhydrous ethanol (Koptec V1016, King of Prussia, PA: 1% solution: 0.1 ml ethanol, 3.18 g sucrose and 7.9 ml water; 2% solution, as 1% solution but with 0.2 ml ethanol and water correspondingly adjusted to 7.8 ml). An ethanol-free sucrose solution was made with 3.18 g of sucrose within 8 ml water. For all mixtures, water and ethanol volumes were added to pre-measured sucrose masses using precision pipettes. Because 200 proof ethanol is constitutively 99.5% ethanol, actual ethanol concentrations are marginally lower than the integer values used here. Presented in mass by mass (m/m) format, these solutions correspond to 0.7% and 1.4% ethanol in 28% sucrose solution, but we used a volume/volume (v/v) format for logistical and presentational convenience, and to enable comparisons with existing data. Estimates of rates of ethanol consumption are, however, calculated using the mass-based values. Experimental solutions were made up weekly and were refrigerated to inhibit fermentation. Anna's Hummingbirds exhibit high preference for aqueous sucrose over glucose or fructose solutions [18], and we accordingly used the former carbohydrate. Ethanol concentrations were in the range of those measured in the mammal-visited flower of a tropical palm (see [5]), but no other relevant data are available to characterize ethanol within naturally occurring nectar.

For binary-choice trials, an individual hummingbird was placed in a plexiglas cube (0.9 × 0.9 × 0.9 m) equipped with a perch equidistant from two suspended 10 ml syringes, each filled with approximately 4 ml of experimental nectar. Syringes were fitted with plastic flowers identical to those used in husbandry cages. The rate of nectar consumption was measured over 3 h by measuring the mass lost from each syringe at the end of each trial; the differential in consumed mass between the two syringes (with either positive or negative value) was then used to indicate preference. Syringe placements within the cube were switched at 90 min to control for potential side-biases. Experimental trials consisted of exposing each bird on different days to a randomly sequenced pair of either 0%, 1% or 2% ethanol solution, such that all pairwise combinations (i.e. 0% versus 1%, 1% versus 2% and 0% versus 2%) completed one experimental series. Each series also included a baseline comparison between two 0% ethanol solutions, for a total of four pairwise comparisons. The full experimental series was then repeated twice per bird over a period of three–four weeks for a total of three series (and thus with three repeats per bird for each pairwise comparison). For one bird, one trial (i.e. 1% versus 2% ethanol) could not be carried out as the bird was released prior to completing the experimental series, and three trials were not carried out for another bird, yielding a total of 32 binary comparisons between different solutions. Two-way factorial ANOVA (Statview 5.0.1, SAS Institute Inc.) was used to assess variation among differential rates of nectar consumption among pairwise presentations of ethanol concentrations, and among individual birds. Fisher's LSD *post hoc* tests were used to assess responses to specific pairwise comparisons.

To assess the extent of ethanolic fermentation within hummingbird feeders (Perky-Pet, Lancaster, PA), we conducted three multi-week experiments in the spring of 2022, one with a 1400 ml top-fill feeder (28-day duration) and two using a 350 ml pinch-waist feeder (each for a 14-day duration). Feeders were filled with 20% sucrose (v/v) in deionized water and were suspended within an open window box on the campus of the University of California, Berkeley, such that wild Anna's Hummingbirds could freely visit (no other trochilid species has been seen at this feeder location over a period of approximately 20 years). Feeders were sampled weekly until most nectar had been consumed (four weeks for the large feeder, and two weeks for the smaller feeders). Nectar samples (approx. 1 ml) were taken from feeder drinking ports using a syringe, then stored at −20°C. Air temperature in the vicinity of the feeder was also measured continuously and averaged on a weekly basis. For analysis, samples were diluted 10-fold and ethanol content was measured using a colorimetric assay based on oxidation of ethanol by alcohol dehydrogenase (EnzyChrom Ethanol Assay Kit ECET-100, BioAssay Systems; detection limit of 0.0008% ethanol). In parallel, calibration assays were carried out using 0%, 0.01%, 0.03% and 0.06% ethanol within 20% sucrose standards (all values in v/v format).

## 3. Results

**Figure 1.**
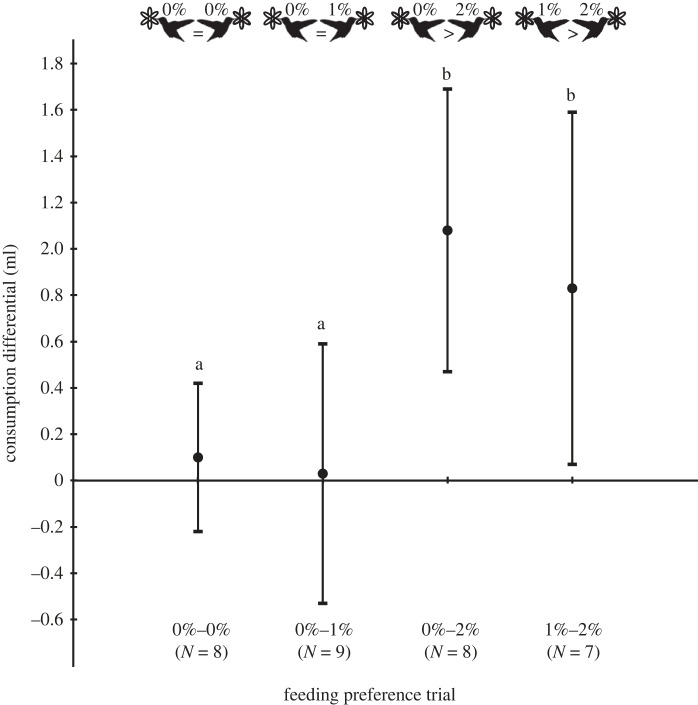
Volumetric consumption differentials for binary choices between experimental nectar solutions over a 3 h interval, with concentration comparisons indicated beneath data points. A positive differential indicates greater consumption of the first of the two indicated concentrations (error bars indicate 1 s.d.); different letters above data points indicate significant differences (see text). Silhouette of *Calypte anna* by Michaud Margot (Creative Commons, http://phylopic.org).

Hummingbirds consumed nectar from each of 0, 1 and 2% ethanol solutions, albeit to varying extent. Overall, the birds were indifferent to the presence of 1% ethanol in nectar relative to a 0% choice test, but reduced their volumetric consumption of 2% ethanol when it was paired with either 0% or 1% ethanol (figure 1). The three hummingbirds responded equivalently to binary choices among presented solutions, showing no statistical differences among individuals in their differential extent of nectar consumption in response to ethanol concentrations in any given binary choice (*F* = 0.25, d.f. = 2, 20; *p* = 0.78). There were also no significant interaction effects between bird identity and the differential extent of nectar consumption between syringes (*F* = 0.46, d.f. = 6, 20; *p* = 0.83). However, there was an overall significant difference in the differential rates of nectar consumption among the four binary-choice comparisons (*F* = 5.82, d.f. = 3, 20; *p* = 0.005; see also figure 1).

*Post hoc* comparisons confirmed aversion to 2% ethanol solutions, such that the consumption differential in the [0%–2%] choice was greater (i.e. higher consumption of the 0% solution) compared to that of the [0%–0%] choice (*p* < 0.006) and of the [0%–1%] choice (*p* < 0.003). Similarly, the consumption differential was significantly greater for the [1%–2%] choice (i.e. higher consumption of the 1% solution) compared to the differential in the [0%–0%] choice (*p* < 0.04) and to that in the [0%–1%] choice (*p* < 0.03).

Overall, hummingbirds across all trials tended to consume about half the volume of the 2% ethanol solution relative to the 1% solution when the alternative was 0% ethanol (table 1), whereas the consumption differential did not vary between the [0%–0%] and [0%–1%] choice trials, indicating that birds were indifferent to the presence of 1% ethanol relative to its absence (*p* > 0.80; figure 1, table 1). Averaged across all three birds, the estimated mass rates of ethanol consumption from the 1% and the 2% solutions (when paired with 0% ethanol) were nearly equivalent (approx. 3.6 mg h^−1^; table 1); consumption rates of nectar and total ethanol across all trials for individual birds are illustrated in electronic supplementary material, figure S1.

**Table 1.** Average hourly rates (range) for total nectar and ethanol consumption in each of four binary-choice tests among combinations of 0%, 1% and 2% ethanol in 20% sucrose solution, as pooled among all corresponding trials for three individual hummingbirds.

	[0%–0%]	[0%–1%]	[0%–2%]	[1%–2%]
nectar (g h^−1^)	0.80 (0.73–0.97)	0.73 (0.64–0.92)	0.72 (0.60–0.86)	0.73 (0.66–0.84)
ethanol (mg h^−1^)	0.0	3.60 (1.73–5.3)	3.57 (1.33–7.07)	9.52 (7.63–11.1)

**Figure 2.**
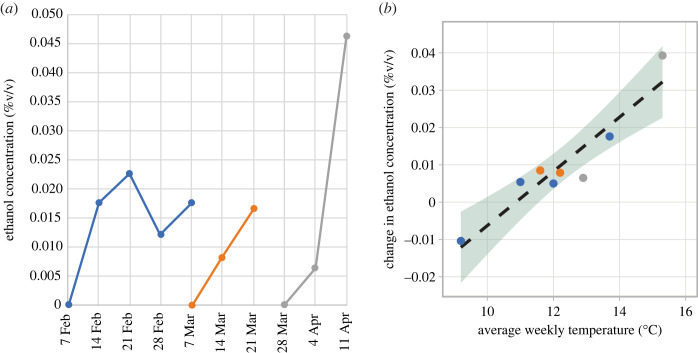
(*a*) Ethanol concentration of hummingbird feeder solutions through time for a large feeder (blue line), and for two trials with a small feeder (orange and grey lines). (*b*) Weekly change in ethanol concentration as a function of weekly mean temperature. The linear regression is given by *y* = 0.007 *x* −0.08 (adj. *R*^2^ = 0.85, *p* < 0.001); shaded regions indicate 95% confidence intervals.

Ethanol concentrations of sucrose solutions within hummingbird feeders were initially zero and then tended to increase with calendar day, peaking for one feeder at approximately 0.045% ethanol v/v (figure 2*a*). Ethanol concentration declined over one week in one feeder, concurrent with a reduction in air temperature over the same period. Combining data for all feeders, the average weekly change in ethanol concentration (either positive or negative) was significantly and positively correlated with average weekly air temperature (figure 2*b*).

## 4. Discussion

Hummingbirds can differentiate among low-concentration ethanol solutions and prefer either 0% or 1% ethanol to 2% ethanol (in 20% sucrose solutions) when presented with a binary choice. Birds nonetheless did consume 2% ethanol at substantial rates, and the dosage was comparable to that consumed from the 1% solution, when either was paired with a 0% solution (table 1). The associated intake (approx. 3.6 mg h^−1^) may thus represent a limiting threshold beyond which toxicological effects can pertain; this condition may have in fact been attained in the [1%–2%] binary-choice tests (i.e. 9.5 mg h^−1^; table 1) during which the birds were unable to avoid ethanol in solution. Hummingbirds can therefore ingest and apparently tolerate low-level ethanol concentrations within nectar, although the specific pathways for absorption and digestion of this molecule have not been described in birds. Key enzymes involved in ethanol catabolism include various alcohol and acetaldehyde dehydrogenases (ADH and ALDH, respectively), but these have not been specifically characterized for birds in relation to their feeding ecology. However, Janiak *et al*. [19] found considerable variation among mammalian species in one particular ADH gene (ADH7), and positive selection on this gene was also found to be higher in those species that regularly consume either fruit or nectar, suggesting that it may facilitate chronic ingestion of fermentation products. Broad genomic comparisons among bird species in ADH and ALDH could provide similar insights into nectarivore physiology and those evolutionary pressures associated with low-level ethanol in the diet.

One limitation of this study is that our experimental nectars do not necessarily replicate naturally fermented nectar, or even those fermenting sucrose solutions likely found in hummingbird feeders. For example, microbial communities within hummingbird feeders contain a variety of fungal and bacterial species spread by bird visitation, and these can differ from those in flowers [17]. Nectarivores may also rely on fermentative yeasts and other microbes as reliable indicators of the presence of sugars or other nutrients (including yeasts themselves). For example, bumblebees prefer nectars with greater yeast content [14], and a variety of microbially derived compounds may influence pollinator–flower interactions [20]. Further investigation into nectar and feeder microbial populations may give insights into additional features of nectar that may influence hummingbird preference, such as pH, sugar concentration and ethanol concentrations exceeding 2%.

Ethanol at 1% is not aversive to hummingbirds, but likely provides minimal energetic benefit relative to that of the commingled sucrose in solution. The measured ingestion rates of 20% sucrose solution (table 1) were only about half those estimated for wild Anna's Hummingbirds (approx. 2 g sucrose daily, assuming a 12 h foraging period; see [21]). Hummingbirds studied here were kept in captivity for up to one month with ad libitum access to nectar and may have had differing activity budgets and net energy balance relative to free-ranging birds. Hummingbird indifference to 1% ethanol is also comparable to results for avian frugivores. Three frugivorous bird species in southern Africa (the Cape White-eye [*Zosterops virens*], Speckled Mousebird [*Colius striatus*] and the Red-winged Starling [*Onychognathus morio*]) exhibited no preference for artificial diets containing 1% ethanol relative to 0% [22], and Yellow-vented Bulbuls (*Pycnonotus xanthopygos*) were indifferent to ethanol concentrations up to 2% [23].

Preference for 1% over 2% ethanol (and for 0% over 2%) was pronounced in Anna's Hummingbirds and was presumably mediated via taste. The amount of ingested ethanol from either 1% or 2% ethanol solutions against a 0% solution (as averaged across all pairwise experimental trials) was however substantial and nearly equivalent, given that the 2% ethanol solution was ingested at nearly one-half the rate of the 1% solution (table 1). The estimated ingestion rate of approximately 3.6 mg ethanol hourly corresponds to a body mass specific rate of ingestion that is close to 0.1%/h. Concentration-dependent aversion to ethanol has been documented for other vertebrates. For example, Egyptian fruit bats (*Rousettus aegyptiacus*) avoid fruit with ethanol concentrations greater than 1% [24], and Yellow-vented Bulbuls exhibit a decreased intake of fruit at ethanol concentrations > 2% [23]. One motivating factor for such selectivity may be the ataxia or even toxicosis that can ensue following high rates of ethanol ingestion (e.g. [25]). Contrariwise, physiological benefits may accrue with chronic consumption of low levels of ethanol. For example, longevity and fecundity of fruit flies increase at intermediate levels of gaseous ethanol exposure [26], and chronic low-level ingestion of ethanol by mice is associated with increased lifespan [27]. For a foraging Anna's Hummingbird, 2% ethanol in nectar may simply not be experienced in the wild and could pose toxicological risk, whereas routine consumption of 1% ethanol (or lower concentrations) could represent the natural ecological background of dietary exposure for which the bird's metabolic capacity is well-suited, and which may provide benefit via hormetic effects. In this case, dietary choice may just track environmental availability; this is an empirically testable hypothesis, and one which may yield different preference thresholds given the wide geographical range of many hummingbird taxa, both intra- and interspecifically.

Measurements on sugar solutions within hummingbird feeders yielded detectable amounts of ethanol, as presumably produced by fermentative yeasts (and possibly by some bacteria). Ethanol concentrations also tended to increase with ambient temperature, and the highest concentration occurred after the warmest week in the study period (figure 2). Although the feeders were new, neither they nor the sugar solution placed therein were sterilized prior to use, so as to simulate actual implementation ‘in the wild’ by humans. Similarly, visitation by hummingbirds (and possibly by insects) was not monitored. Furthermore, we did not measure nectar temperatures, which may have been elevated via microbial activity above ambient air temperature and which could have influenced yeast growth and fermentation. The microbial fauna and dynamics of anthropogenic nectar sources are clearly complex, but deserve further attention given their widespread use and possible supplementation of the hummingbird diet with non-sugar components such as ethanol.

Ethanol concentrations within feeders, although seemingly low, were however non-trivial given high volumetric rates of nectar consumption by hummingbirds. Anna's Hummingbirds must consume about 10 g of 20% sucrose solution daily to meet energetic needs [21]. Even if typical ethanol concentrations in feeders are only 0.02%, such a rate of consumption would still correspond to a daily dosage of 2 mg of ethanol (or about 0.04% of body mass). By contrast, human consumption of one standard drink daily (containing 12 g of ethanol) corresponds to a exposure of about 0.015% of body mass, assuming the latter to be 80 kg.

Floral nectar starts to ferment as soon as it is exposed to microbes, particularly yeasts. Thus, the occurrence of unfermented nectar in nature may well be rare given the apparent ubiquity of nectarivorous yeasts. For example, a large fraction of South African flowers contained such microbes at considerable volumetric densities, with bird-pollinated taxa showing a higher incidence relative to insect-pollinated flowers [28]. Comparative studies of ethanol generation within nectar, particularly for those flowers frequented by hummingbirds and other nectar-feeding birds, are needed to place these experimental results within a broader ecological context. Nectarivore responses to ethanol are likely to derive from numerous factors, including interspecific variation in the ability to metabolize ethanol and variation in availability of ethanol within floral nectar. This latter feature may derive from differences in microbial communities, sugar concentrations, and fermentation rates relative to evaporative loss, and microclimatic along with larger scale temperature regimes for particular floral habitats.

## 5. Conclusion

Indifference by Anna's Hummingbirds to 1% ethanol sugar solutions, along with still substantial rates of ethanol ingestion at the less-preferred 2% solution, indicates the possibility of chronic exposure via floral nectar as well as from anthropogenic feeders. Discrimination among low-concentration ethanol solutions by hummingbirds also suggests that the ability to sense ethanol, and consequently to alter feeding responses, may be widespread among the many bird taxa that consume nectar. The ecological consequences of dietary ethanol exposure, including effects on plant–pollinator interactions (as well as longer term health consequences for nectarivores) may thus be substantial, given the ubiquity of fermenting microbes within naturally occurring sugary foods.

## Data Availability

Associated data files are available at the following link: https://doi.org/10.6078/D1WM6T [29]. The data are provided in the electronic supplementary material [30].

## References

[1] Raubenheimer D, Simpson S. 1997 Integrative models of nutrient balancing: application to insects and vertebrates. Nutr. Res. Rev. **10**, 151-179. (10.1079/NRR19970009) 19094262

[2] Demi LM, Taylor BW, Reading BJ, Tordoff MG, Dunn RR. 2021 Understanding the evolution of nutritive taste in animals: insights from biological stoichiometry and nutritional geometry. Ecol. Evol. **11**, 8441-8445. (10.1002/ece3.7745) 34257909 PMC8258225

[3] Whiteman N. 2023 Most delicious poison: the story of nature's toxins—from spices to vices. Boston, MA: Little Brown, Spark.

[4] Dudley R. 2014 The drunken monkey: why we drink and abuse alcohol. Berkeley, CA: University of California Press.

[5] Wiens F, Zitzmann A, Lachance MA, Yegles M, Pragst F, Wurst FM, von Holst D, Guan SL, Spanagel R. 2008 Chronic intake of fermented floral nectar by wild treeshrews. Proc. Natl Acad. Sci. USA **105**, 10 426-10 431. (10.1073/pnas.0801628105) PMC2492458 18663222

[6] Gochman SR, Brown MB, Dominy NJ. 2016 Alcohol discrimination and preferences in two species of nectar-feeding primate. R. Soc. Open Sci. **3**, 160217. (10.1098/rsos.160217) 27493777 PMC4968469

[7] Ibañez DD, Salazar LT, Laska M. 2019 Taste responsiveness of spider monkeys to dietary ethanol. Chem. Senses **44**, 631-638. (10.1093/chemse/bjz049) 31400282

[8] Peris JE, Rodríguez A, Peña L, Fedriani JM. 2017 Fungal infestation boosts fruit aroma and fruit removal by mammals and birds. Sci. Rep. **7**, 1-9. (10.1038/s41598-017-05643-z) 28717123 PMC5514155

[9] Campbell C, Maro A, Weaver V, Dudley R. 2022 Dietary ethanol ingestion by free- ranging spider monkeys (*Ateles geoffroyi*). R. Soc. Open Sci. **9**, 211729. (10.1098/rsos.211729) 35345427 PMC8941420

[10] Janzen DH. 1977 Why fruits rot, seeds mold, and meat spoils. Am. Nat. **111**, 691-713. (10.1086/283200)

[11] Fleming TH, Kress WJ. 2013 The ornaments of life: coevolution and conservation in the tropics. Chicago, IL: University of Chicago Press.

[12] Hewes AE, Cuban D, Groom DJE, Sargent AJ, Beltrán DF, Rico-Guevara A. 2022 Variable evidence for convergence in morphology and function across avian nectarivores. J. Morph. **283**, 1483-1504. (10.1002/jmor.21513) 36062802

[13] Lievens B, Hallsworth JE, Pozo MI, Belgacem ZB, Stevenson A, Willems KA, Jacquemyn H. 2015 Microbiology of sugar-rich environments: diversity, ecology and system constraints. Environ. Microbiol. **17**, 278-298. (10.1111/1462-2920.12570) 25041632

[14] Schaeffer RN, Mei YZ, Andicoechea J, Manson JS, Irwin RE. 2016 Consequences of a nectar yeast for pollinator preference and performance. Funct. Ecol. **31**, 613-621. (10.1111/1365-2435.12762)

[15] Dzialo MC, Park R, Steensels J, Lievens B, Verstrepen KJ. 2017 Physiology, ecology, and industrial applications of aroma formation in yeast. FEMS Microbiol. Rev. **41**, S95-S128. (10.1093/femsre/fux031) 28830094 PMC5916228

[16] Rering CC, Jeck JJ, Hall GW, Mitchell M, McCartney MM, Vannette RL. 2018 Nectar-inhabiting microorganisms influence nectar volatile composition and attractiveness to a generalist pollinator. New Phytol. **220**, 750-759. (10.1111/nph.14809) 28960308

[17] Lee C, Tell LA, Hilfer T, Vannette RL. 2019 Microbial communities in hummingbird feeders are distinct from floral nectar and influenced by bird visitation. Proc. R. Soc. Lond. B **286**, 20182295. (10.1098/rspb.2018.2295) PMC6458324 30836877

[18] Stiles FG. 1976 Taste preferences, color preferences, and flower choice in hummingbirds. The Condor **78**, 10-26. (10.2307/1366912)

[19] Janiak MC, Pinto SL, Duytschaever G, Carrigan MA, Melin AD. 2020 Genetic evidence of widespread variation in ethanol metabolism among mammals: revisiting the ‘myth’ of natural intoxication. Biol. Lett. **16**, 20200070. (10.1098/rsbl.2020.0070) 32343936 PMC7211468

[20] Martin VN, Schaeffer RN, Fukami T. 2022 Potential effects of nectar microbes on pollinator health. Phil. Trans. R. Soc. Lond. B **377**, 20210155. (10.1098/rstb.2021.0155) 35491594 PMC9058548

[21] Powers DR, Nagy KA. 1988 Field metabolic rate and food consumption by free-living Anna's hummingbirds (*Calypte anna*). Physiol. Zool. **61**, 500-506. (10.1086/physzool.61.6.30156158)

[22] Zungu MM, Downs CT. 2017 Effects of ethanol on fruit selection by frugivorous birds. Afr. Zool. **52**, 69-72. (10.1080/15627020.2016.1276856)

[23] Mazeh S, Korine C, Pinshow B, Dudley R. 2008 The influence of ethanol on feeding in the frugivorous yellow-vented bulbul (*Pycnonotus xanthopygos*). Behav. Proc. **77**, 369-375. (10.1016/j.beproc.2007.10.003) 18024002

[24] Sánchez F, Korine C, Steeghs M, Laarhoven LJ, Cristescu SM, Harren FJM, Dudley R, Pinshow B. 2006 Ethanol and methanol as possible odor cues for Egyptian fruit bats (*Rousettus aegyptiacus*). J. Chem. Ecol. **32**, 1289-1300. (10.1007/s10886-006-9085-0) 16770719

[25] Kinde H, Foate E, Beeler E, Uzal F, Moore J, Poppenga R. 2012 Strong circumstantial evidence for ethanol toxicosis in Cedar Waxwings (*Bombycilla cedrorum*). J. Ornithol. **153**, 995-998. (10.1007/s10336-012-0858-7)

[26] Parsons PA, Spence GE. 1981 Longevity, resource utilization and larval preferences in *Drosophila*: inter-and intraspecific variation. Aust. J. Zool. **29**, 671-678.

[27] Diao Y et al. 2020 Long-term low-dose ethanol intake improves healthspan and resists high-fat diet-induced obesity in mice. Aging **12**, 13 128-13 146. (10.18632/aging.103401) PMC7377878 32639947

[28] DeVega C, Herrera CM, Johnson CD. 2009 Yeasts in floral nectar of some South African plants: quantification and associations with pollinator type and sugar concentration. S. Afr. J. Sci. **75**, 798-806. (10.1016/j.sajb.2009.07.016)

[29] Choi J, Lee L, Maro A, Corl A, McGuire JA, Bowie RCK, Dudley R. 2023 Supplementary data for: Hummingbird ingestion of low-concentration ethanol within artificial nectar. Dryad, Dataset. (10.6078/D1WM6T) PMC10282586 37351493

[30] Choi J, Lee L, Maro A, Corl A, McGuire JA, Bowie RCK, Dudley R. 2023 Hummingbird ingestion of low-concentration ethanol within artificial nectar. Figshare. (10.6084/m9.figshare.c.6697362) PMC10282586 37351493

